# Nosocomial transmission of fluconazole-resistant *Candida glabrata* bloodstream isolates revealed by whole-genome sequencing

**DOI:** 10.1128/spectrum.00883-24

**Published:** 2024-08-20

**Authors:** In Ji Hwang, Yong Jun Kwon, Ha Jin Lim, Ki Ho Hong, Hyukmin Lee, Dongeun Yong, Eun Jeong Won, Seung A. Byun, Ga Yeong Lee, Soo Hyun Kim, Eun Song Song, Jong Hee Shin

**Affiliations:** 1 Department of Pediatrics, Chonnam National University Medical School, Gwangju, South Korea; 2 Department of Laboratory Medicine, Chonnam National University Medical School, Gwangju, South Korea; 3 Department of Laboratory Medicine and Research Institute of Bacterial Resistance, Yonsei University College of Medicine, Seoul, South Korea; 4 Department of Laboratory Medicine, Asan Medical Center, University of Ulsan College of Medicine, Seoul, South Korea; Central Texas Veterans Health Care System, Temple, Texas, USA

**Keywords:** *Candida glabrata*, whole-genome sequencing, clonal transmission, fluconazole resistance, PDR1, multilocus sequence typing

## Abstract

The clonal transmission of fluconazole-resistant *Candida glabrata* isolates within hospitals has seldom been analyzed by whole-genome sequencing (WGS). We performed WGS on 79 *C*. *glabrata* isolates, comprising 31 isolates from three premature infants with persistent *C. glabrata* bloodstream infection despite antifungal treatment in the same neonatal intensive care unit (NICU) in 2022 and 48 (27 fluconazole-resistant and 21 fluconazole-susceptible dose-dependent) bloodstream isolates from 48 patients in 15 South Korean hospitals from 2010 to 2022. Phylogenetic analysis based on WGS single-nucleotide polymorphisms (SNPs) distinguished the 79 isolates according to multilocus sequence typing (MLST) (17 sequence type [ST]3, 13 ST7, two ST22, 41 ST26, four ST55, and two ST59 isolates) and unveiled two possible clusters of nosocomial transmission among ST26 isolates. One cluster from two premature infants with overlapping NICU hospitalizations in 2022 encompassed 15 fluconazole-resistant isolates harboring pleiotropic drug-resistance transcription factor (Pdr1p) P258L (13 isolates) or N1086I (two isolates), together with 10 fluconazole-susceptible dose-dependent isolates lacking Pdr1p SNPs. The other cluster indicated unforeseen clonal transmission of fluconazole-resistant bloodstream isolates among five patients (four post-lung transplantation and one with diffuse interstitial lung disease) in the same hospital over 8 months. Among these five isolates, four obtained after exposure to azole antifungals harbored distinct Pdr1p SNPs (N1091D, E388Q, K365E, and R376Q). The findings reveal the transmission patterns of clonal bloodstream isolates of *C. glabrata* among patients undergoing antifungal treatment, exhibiting different levels of fluconazole susceptibility or distinct Pdr1p SNP profiles.

**IMPORTANCE:**

The prevalence of fluconazole-resistant bloodstream infections caused by *Candida glabrata* is increasing globally, but the transmission of these resistant strains within hospitals has rarely been documented. Through whole-genome sequencing and epidemiological analyses, this study identified two potential clusters of *C. glabrata* bloodstream infections within the same hospital, revealing the transmission of clonal *C. glabrata* strains with different levels of fluconazole susceptibility or distinct transcription factor pleiotropic drug resistance protein 1 (Pdr1p) single-nucleotide polymorphism profiles among patients receiving antifungal therapy.

## INTRODUCTION

*Candida* bloodstream infection (BSI) is the most common life-threatening fungal disease in hospitalized patients (1–3). *Candida glabrata,* recently renamed *Nakaseomyces glabrata* (4), is the second-most frequent cause of *Candida* BSIs following *Candida albicans* in many countries (5–7). *C. glabrata* exhibits innately low susceptibility to azole drugs, especially fluconazole, and can rapidly develop acquired resistance to azole or echinocandin during antifungal treatment (3, 5, 6). Although the incidences of echinocandin- and multidrug-resistant (MDR) *C. glabrata* BSIs are low, the incidence of fluconazole-resistant (FR) *C. glabrata* BSI is increasing worldwide (3, 5, 6, 8). Gain-of-function mutations in the transcription factor pleiotropic drug resistance protein 1 (encoded by *PDR1*) mediate fluconazole resistance in *C. glabrata* by controlling the expression of genes encoding efflux pumps, such as *CDR1, CDR2* (*PDH1*), and *SNQ2*; other mechanisms may also contribute to azole resistance (6, 8–12). Due to the intrinsically low susceptibility to fluconazole, *C. glabrata* isolates are no longer considered susceptible to fluconazole but are categorized as fluconazole-susceptible dose-dependent (F-SDD) or FR (13). Fluconazole resistance in *C. glabrata* is of particular concern because of the increased incidence of BSIs caused by this species in a variety of locations worldwide, and it is now often accompanied by echinocandin resistance, resulting in the emergence of MDR strains (3, 5, 6, 14). More importantly, a high mortality rate of patients infected with FR *C glabrata* BSI isolates has been reported (2, 8).

The clonal spread of FR *Candida* species such as *Candida parapsilosis* or *Candida auris* has been reported in hospitals worldwide (6, 15–20). Whole-genome sequencing (WGS) has frequently been utilized to track the development and clonal transmission of FR *C. parapsilosis* or *C. auris* within healthcare facilities (16–20). Although less common than other *Candida* species, the potential for intra- or interhospital transmission of *C. glabrata* has been reported, with varying levels of certainty (21–24). However, despite the increasing number of cases of BSIs caused by FR *C. glabrata* globally (3, 5, 6, 8), the contribution of the clonal transmission of FR *C. glabrata* to the increases in BSI cases is unclear. The lack of reports of clonal transmission of FR *C. glabrata* BSI strains may be partly due to the infrequent occurrence of nosocomial transmission of FR strains, the underutilization of dependable genotyping tools, or the frequent genomic changes in haploid *C. glabrata in vivo* (23, 24). WGS has recently been employed to analyze *C. glabrata* infections to analyze potential antifungal resistance mechanisms and to determine the possibility of intra- or inter-hospital transmission of *C. glabrata* isolates (10, 24–27). Nevertheless, few studies have utilized WGS to detect the nosocomial transmission of clonal FR *C. glabrata* bloodstream isolates within healthcare facilities (21–24).

We recently discovered three unusual cases of *C. glabrata* BSI in a neonatal intensive care unit (NICU) in 2022. Despite treatment with antifungal agents such as fluconazole, amphotericin B, or echinocandins, the three premature infants had persistent *C. glabrata* BSI for 51, 56, and 78 days, respectively. The *C. glabrata* isolates from blood, stool, and catheter cultures of these patients included both F-SDD and FR isolates. In this study, we investigated the genetic relatedness of sequential clinical isolates of *C. glabrata* from three premature infants in a single NICU during the same time period in 2022 to trace the possible transmission patterns of clonal FR *C. glabrata* isolates via WGS. The 48 unrelated *C. glabrata* BSI isolates (27 FR and 21 F-SDD) strains obtained from blood cultures of 48 patients spanning a 12-year period (2010–2022) across 15 Korean hospitals nationwide were included as control strains, as routine hospital surveillance did not detect the evidence of clonal transmission in all of these isolates. Furthermore, we investigated the possible molecular mechanisms of antifungal resistance by examining the potential genetic markers for antifungal resistance or underlying genetic factors through WGS analysis among these 79 isolates of *C. glabrata*.

## RESULTS

### WGS and MLST genotypes

**Fig 1:**
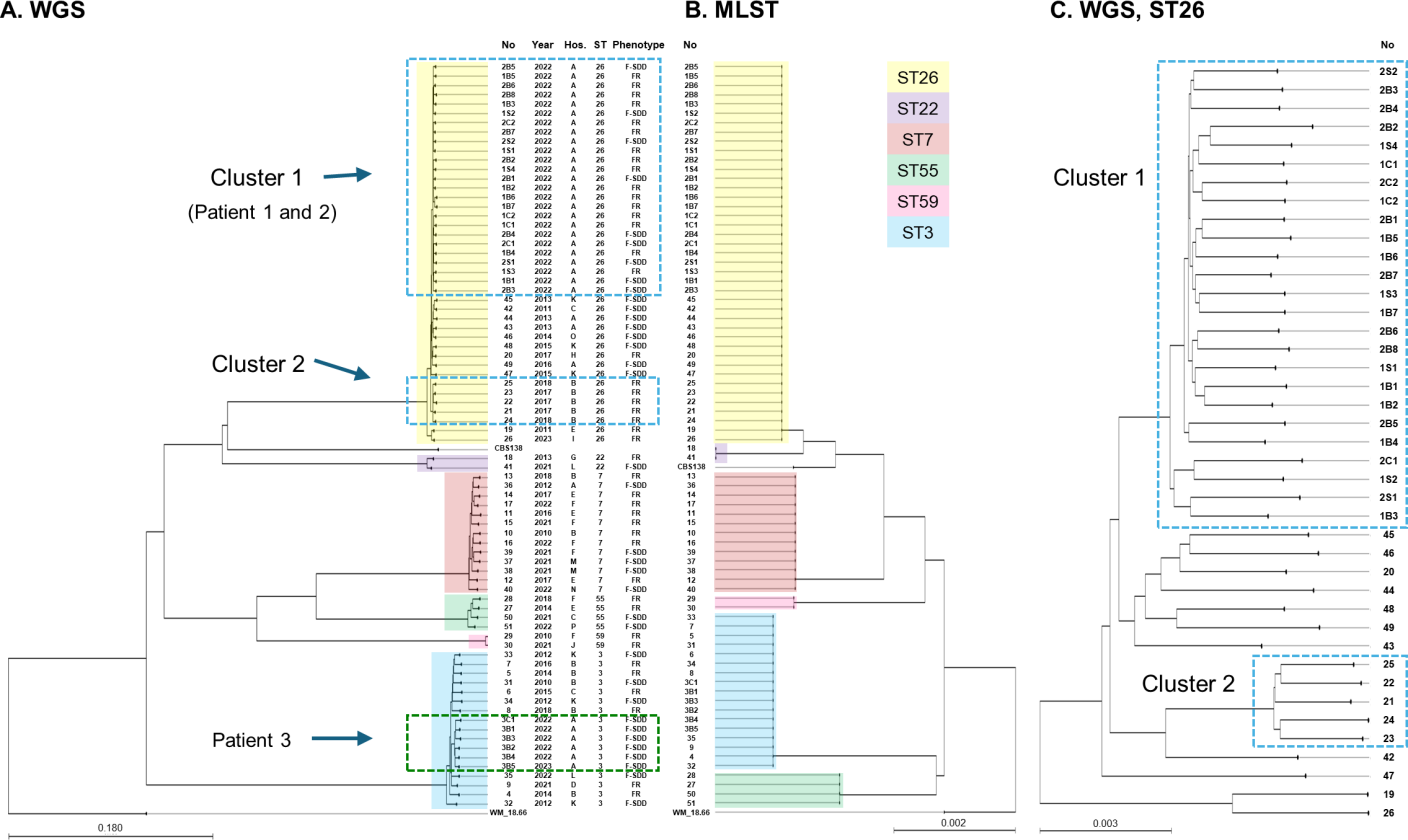
Three phylogenetic trees constructed to analyze the genetic relatedness among *C. glabrata* isolates. The first tree (**A**) was constructed using the average nucleotide identity-based neighbor-joining method of 12,338,304 nucleotide positions from whole-genome sequencing (WGS) analysis of 79 *C*. *glabrata* isolates from 51 patients (Patients 1–51). The second tree (**B**) was based on single-nucleotide polymorphisms (SNPs) from six multilocus sequencing typing (MLST) loci for the same 79 isolates. Finally, a WGS-based phylogenetic tree (**C**) was generated to show the genetic relatedness among 41 *C*. *glabrata* isolates from 18 patients with ST26. Information is shown in the format isolate number (No), isolation year, hospital (Hos A–O), MLST sequence type (ST), and phenotype (FR, fluconazole-resistant; F-SDD, fluconazole-susceptible dose-dependent). The isolate numbers of three premature patients (Patients 1, 2, and 3) in the same ICU include the patient number and the serial number of the source (B, blood; C, central venous catheter tip; S, stool). Two blue boxes with dotted lines in ST26 indicate the two nosocomial clusters, and the green box with dotted lines in ST3 indicates serial isolates from the same patient (Patient 3). Lower bars indicate genetic distances. See Tables 1 and 2 for information on the isolates.

Figure 1 shows a phylogenetic tree constructed with the average nucleotide identity (ANI)-based neighbor-joining (NJ) method of 12,338,304 nucleotide positions in WGS data for 79 *C*. *glabrata* isolates from 51 patients (Patients 1–51). These 79 isolates of *C. glabrata* were separated according to their MLST sequence types (17 sequence type [ST]3, 13 ST7, two ST22, 41 ST26, four ST55, and two ST59 isolates). After comparing the phylogenetic relationships among strains with their epidemiological information, such as hospital (A to O) or isolation year (2010 to 2022) in individual clusters, we detected the two possible nosocomial clusters within isolates of ST26 (Clusters 1 and 2). Cluster 1 consisted of 25 isolates from two premature infants (Patients 1 and 2) in the same NICU of hospital A in 2022 and included 15 FR isolates and 10 F-SDD isolates. Cluster 2 isolates consisted of five FR BSI isolates obtained from five patients (Patients 21–25) in hospital B during 2017 and 2018. Among 31 serial isolates from three infants admitted to the same NICU, 25 isolates from two NICU infants (Patients 1 and 2) formed a cluster (Cluster 1), which did not differentiate between two patients, while all six serial isolates from the remaining NICU infant (Patient 3) formed a clearly defined cluster within isolates of ST3 in phylogenetic analysis. Figure 1B shows a secondary phylogenetic tree based on six MLST loci and corresponding STs, which was concordant with the trees obtained by WGS. A WGS-based phylogenetic tree showing the genetic relatedness among 41 *C*. *glabrata* isolates from 18 patients of ST26 indicated that WGS data clustered broadly within the same MLST types but showed greater intra-ST resolution (Fig. 1C).

**Fig 2:**
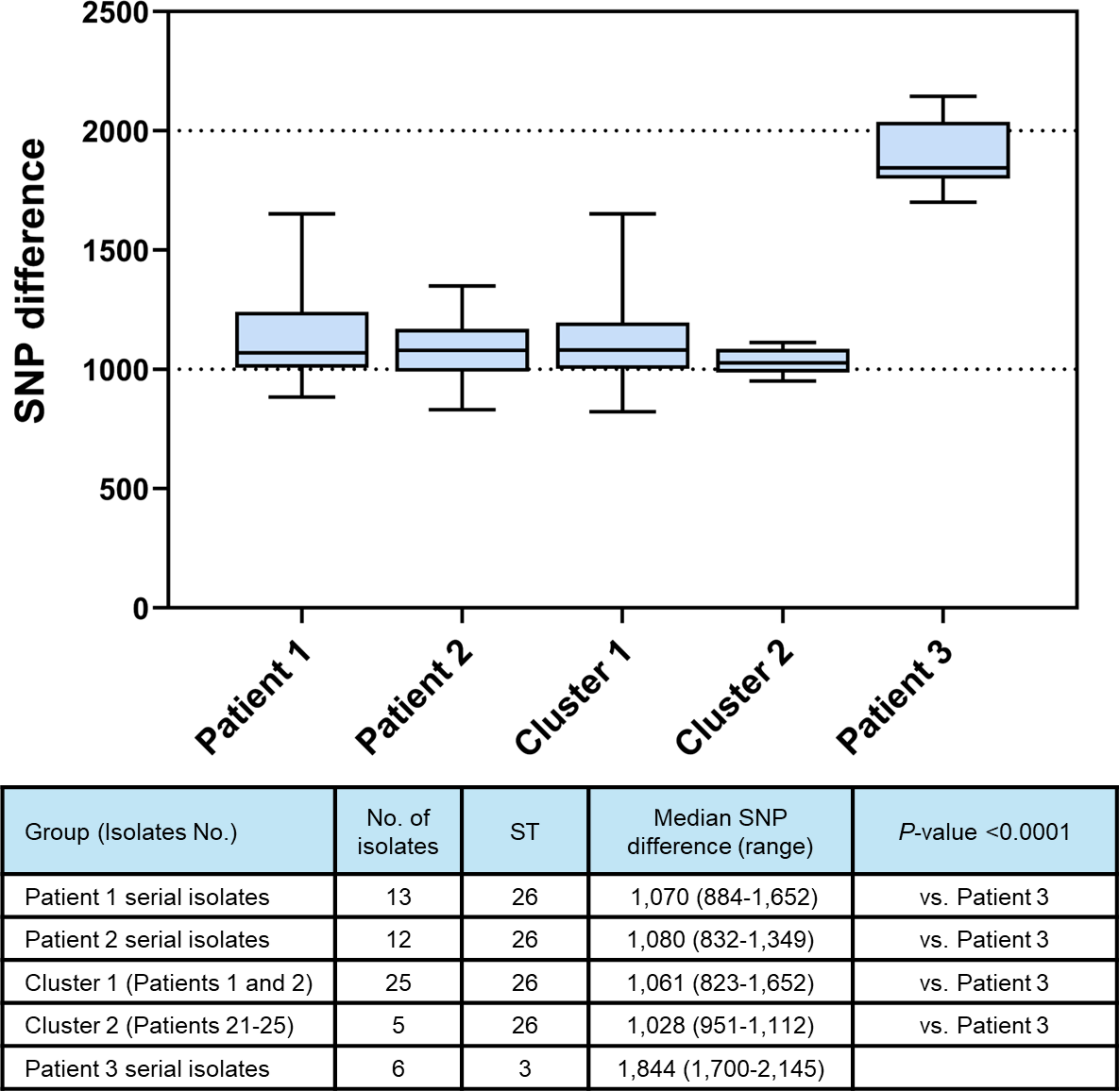
Single-nucleotide polymorphism (SNP) differences for each pairwise comparison among related isolates, indicating serial isolates from the same patients (Patients 1, 2, and 3) and each clonal cluster (Clusters 1 and 2). The median SNP differences between isolates for each clonal cluster (Cluster 1 and 2) were significantly lower than the median SNP differences between pairs of six serial isolates of ST3 from Patient 3 (*P*  <  0.0001). Horizontal bars indicate median SNP levels of each group. See Fig. S1 for SNP difference data in detail.

The median SNP differences between isolates from Patient 1 were 1,070 (range, 884–1,652) SNPs, and those from Patient 2 were 1,080 (range, 832–1,349) SNPs (Fig. 2; Fig. S1). The median SNP differences between isolates from clusters 1 and 2 were 1,061 (range, 823–1,652 SNPs) and 1,028 (range, 951–1,112 SNPs), respectively, similar to the median SNP differences between isolates from Patient 1 and Patient 2. However, these four values of median SNP differences between isolates of ST26 were significantly lower than the median SNP difference between pairs of six serial isolates of ST3 from Patient 3 (1,844, *P*  <  0.0001). Of the 182 genes potentially associated with antifungal resistance examined, 128 had the same SNPs in all isolates of the same ST tested, irrespective of their antifungal susceptibility (designated MLST-specific SNPs) (Table S1). Table S2 lists the MLST-specific SNPs in the major antifungal resistance-associated genes found in six STs of *C. glabrata*.

### Comparison of isolates from blood and other sites in NICU patients

Table 1 lists the source and isolation date, antifungal susceptibility data, and the genetic characteristics obtained through WGS for 31 consecutive isolates obtained from three premature infants (Patients 1, 2, and 3). Twenty-five isolates including 13 (7 blood, two catheter, and four stool) from Patient 1 and 12 (8 blood, two catheter, and two stool) from Patient 2 were obtained between January 2022 and March 2022, while all six serial isolates from the remaining NICU infant (Patient 3) were obtained between October 2022 and January 2023. Of 25 isolates from Patients 1 and 2, 15 isolates (10 from Patient 1 and 5 from Patient 2) were FR. These 15 FR isolates were also non-wild-type for voriconazole, posaconazole, and itraconazole, and all harbored Pdt1p SNPs, which were exclusively observed in FR isolates (FR-specific Pdt1p SNPs), after excluding MLST genotype-specific Pdr1p SNPs, as shown in Table S2. Among the 15 FR isolates, one stool isolate from Patient 1 (1S4) and one blood isolate from Patient 2 (2B2) harbored the Pdr1p N1086I mutation; the other 13 FR isolates harbored the Pdr1p P258L mutation. In contrast, the 10 isolates classified as F-SDD (three from Patient 1 and seven from Patient 2) were susceptible to the three azoles, and none exhibited FR-specific Pdr1p SNPs. All six serial isolates from the remaining NICU infant (Patient 3) were susceptible to the three azoles, and none exhibited FR-specific Pdr1p SNPs. None of the 31 isolates from three patients showed resistance to amphotericin B, echinocandins, or 5-fluorocytosine (5-FC), and there were no SNPs in the genes associated with echinocandin resistance (*FKS1* and *FKS2*), 5-FC resistance (*FCY1*, *FCY2*, *FPS1*, and *FPS2*), *MSH*, or representative *ERG* genes (*ERG1*–*ERG11*, *ERG13*, *ERG20*, and *ERG25*–*ERG27*) in any of the isolates.

**TABLE 1:** Antifungal susceptibilities and whole-genome sequence-based molecular characteristics of 31 serial isolates of *C. glabrata* from three premature infants admitted to the same neonatal intensive care unit^*d*^

Patient No	Isolate no^*a*^	Source (isolation date, month/day/year)	Antifungal minimum inhibitory concentrations (ug/mL)^*b*^								Results of whole-genome sequencing^*c*^				
			FLU	VRC	POS	ITR	AMB	ANF	CAF	MCF	MLST (ST)	Pdr1p	Fks1p	Fks2p	Msh2p
1	1B1	Blood (1/12/2022)	32	1	2	1	1	0.03	0.06	0.015	26	None	None	None	None
	1B2	Blood (1/27/2022)	**256**	**8**	**>8**	**>16**	1	0.06	0.12	0.03	26	P258L	None	None	None
	1B3	Blood (2/7/2022)	**256**	**8**	**>8**	**>16**	1	0.12	0.25	0.03	26	P258L	None	None	None
	1B4	Blood (2/14/2022)	**256**	**8**	**>8**	**>16**	1	0.12	0.25	0.03	26	P258L	None	None	None
	1B5	Blood (2/21/2022)	**256**	**8**	**>8**	**>16**	1	0.06	0.12	0.03	26	P258L	None	None	None
	1B6	Blood (2/24/2022)	**256**	**8**	**>8**	**>16**	1	0.12	0.5	0.03	26	P258L	None	None	None
	1B7	Blood (2/28/2022)	**256**	**8**	**>8**	**>16**	1	0.06	0.12	0.03	26	P258L	None	None	None
	1C1	Catheter (2/10/2022)	**256**	**8**	**>8**	**>16**	1	0.12	0.25	0.03	26	P258L	None	None	None
	1C2	Catheter (2/28/2022)	**256**	**8**	**8**	**16**	1	0.12	0.25	0.03	26	P258L	None	None	None
	1S1	Stool (2/16/2022)	32	0.5	1	1	1	0.03	0.06	0.015	26	None	None	None	None
	1S2	Stool (2/16/2022)	32	0.5	1	1	1	0.03	0.06	0.015	26	None	None	None	None
	1S3	Stool (2/16/2022)	**256**	**8**	**>8**	**>16**	1	0.06	0.25	0.03	26	P258L	None	None	None
	1S4	Stool (2/16/2022)	**>256**	**>8**	**>8**	**>16**	1	0.03	0.06	0.015	26	N1086I	None	None	None
2	2B1	Blood (2/4/2022)	16	0.5	2	1	1	0.03	0.06	0.015	26	None	None	None	None
	2B2	Blood (2/4/2022)	**256**	**8**	**>8**	**>16**	1	0.06	0.25	0.015	26	N1086I	None	None	None
	2B3	Blood (2/7/2022)	16	0.5	1	1	1	0.03	0.12	0.015	26	None	None	None	None
	2B4	Blood (2/20/2022)	32	0.5	1	1	1	0.03	0.06	0.015	26	None	None	None	None
	2B5	Blood (2/24/2022)	32	1	2	1	1	0.03	0.06	0.015	26	None	None	None	None
	2B6	Blood (2/28/2022)	**256**	**8**	**>8**	**>16**	1	0.06	0.12	0.015	26	P258L	None	None	None
	2B7	Blood (3/10/2022)	**256**	**8**	**>8**	**>16**	2	0.12	0.25	0.03	26	P258L	None	None	None
	2B8	Blood (3/26/2022)	**256**	**8**	**>8**	**>16**	1	0.06	0.12	0.015	26	P258L	None	None	None
	2C1	Catheter (2/12/2022)	32	0.5	1	1	1	0.03	0.06	0.015	26	None	None	None	None
	2C2	Catheter (2/28/2022)	**256**	**8**	**>8**	**>16**	1	0.12	0.12	0.03	26	P258L	None	None	None
	2S1	Stool (2/16/2022)	16	0.5	1	1	1	0.015	0.06	0.015	26	None	None	None	None
	2S2	Stool (2/16/2022)	32	0.5	2	1	1	0.06	0.12	0.015	26	None	None	None	None
3	3B1	Blood (10/20/2022)	16	0.5	2	1	1	0.03	0.12	0.015	3	None	None	None	None
	3B2	Blood (11/7/2022)	32	1	2	1	1	0.03	0.12	0.015	3	None	None	None	None
	3B3	Blood (11/21/2022)	16	0.5	1	0.5	1	0.06	0.12	0.015	3	None	None	None	None
	3B4	Blood (12/22/2022)	32	1	2	1	1	0.03	0.12	0.015	3	None	None	None	None
	3B5	Blood (1/5/2023)	16	0.5	1	0.5	0.5	0.015	0.06	0.015	3	None	None	None	None
	3C1	Catheter (11/30/2022)	16	0.5	1	1	1	0.06	0.12	0.015	3	None	None	None	None

^*a*^ Isolate number is the patient number and the serial number of the source (B, blood; C, central venous catheter tip; S, stool).

^*b*^ Antifungal minimum inhibitory concentrations determined using the Sensititre Yeast One (SYO) system (Thermo-Scientific, Cleveland, OH, USA). The antifungal resistance category (fluconazole or three echinocandins) and non-wild-type (voriconazole, posaconazole, itraconazole, and amphotericin B) were identified using the Clinical and Laboratory Standards Institute (CLSI) M60-ED (28) and the proposed SYO-specific epidemiological cutoff values (29), respectively. Bold indicates antifungal-resistant or non-wild-type isolates.

^*c*^ SNP data of Pdr1p, Fks1p, and Fks2p were obtained after eliminating MLST genotype-specific SNPs (Table S2). There were no SNPs in the genes associated with 5-FC resistance (*FCY1*, *FCY2*, *FPS1*, and *FPS2*) or representative *ERG* genes (*ERG1–ERG11*, *ERG13*, *ERG20*, and *ERG25–ERG27*) in any of the isolates.

^*d*^ FLU, fluconazole; VRC, voriconazole; POS, posaconazole, ITR, itraconazole; AMB, amphotericin B, ANF, anidulafungin; CAF, caspofungin; MCF, micafungin; MLST, multilocus sequence typing; ST, sequence type.

**Fig 3:**
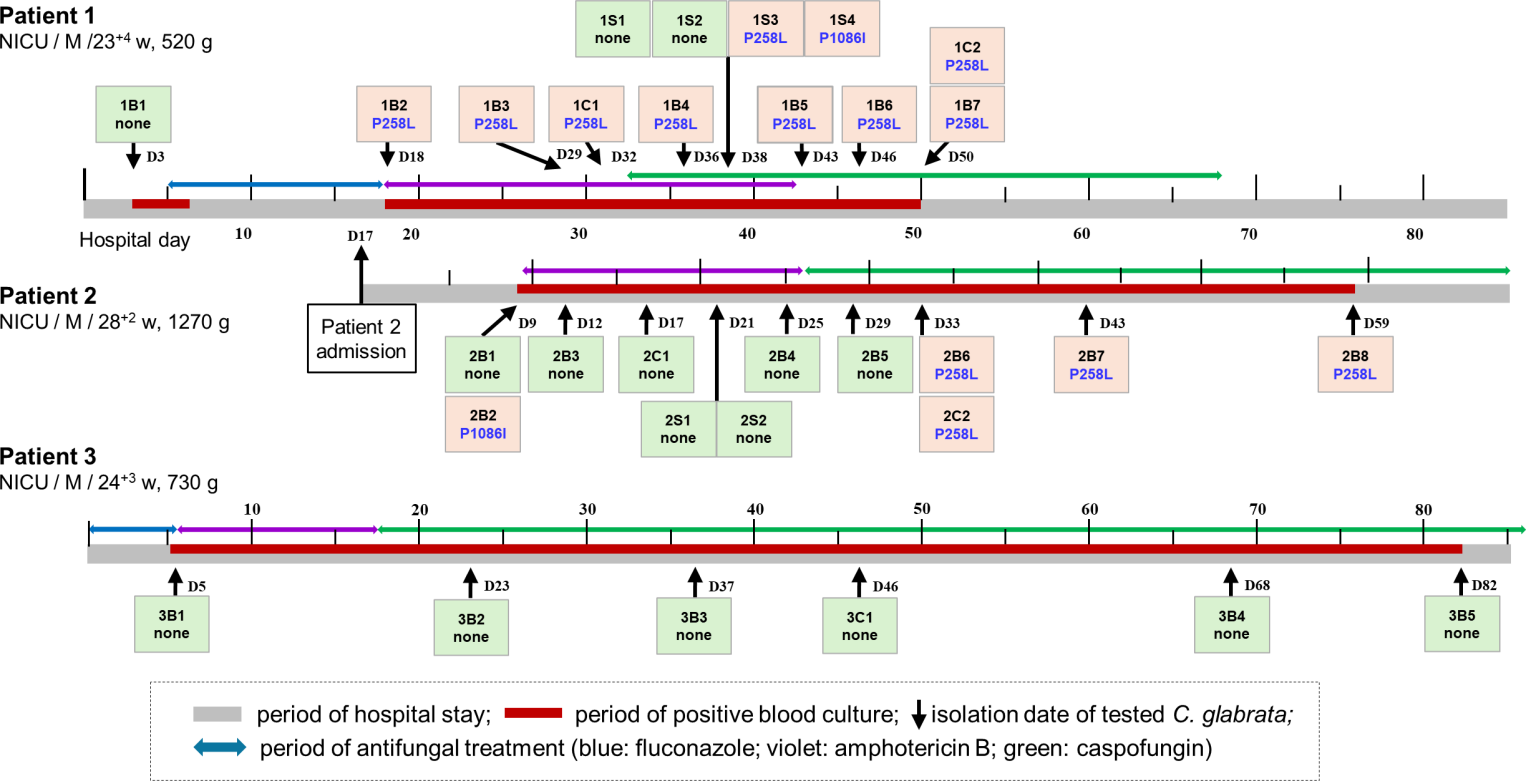
Fluconazole resistance and Pdr1p single-nucleotide polymorphism (SNP) profiles of 31 isolates of *C. glabrata* from three premature infants (Patients 1, 2, and 3) who were admitted to the same NICU. Two patients (Patients 1 and 2) had overlapping hospitalization periods. The boxes contain the isolate numbers with corresponding Pdr1p SNPs for 15 fluconazole-resistant (red boxes) and 16 F-SDD (green boxes) isolates. The isolate numbers of three patients (Patients 1, 2, and 3) include the patient number and the serial number of the source (B, blood; C, central venous catheter tip; S, stool). Table 1 shows information on the isolates. Abbreviations: NICU, neonatal intensive care unit; M, male; w, week.

Figure 3 shows the fluconazole resistance and Pdrp1 SNPs in 31 serial isolates of *C. glabrata* from three NICU patients during antifungal treatment. The first blood isolate from Patient 1 (isolate 1B1) was F-SDD. However, the second BSI isolate (1B2) showed FR and harbored the Pdr1p P258L mutation. Furthermore, the five subsequent BSI isolates and two catheter isolates collected between days 29 and 50 from Patient 1 exhibited FR and harbored Pdr1p P258L. Of the four stool *C. glabrata* isolates obtained on day 38, one displayed FR with Pdr1p P258L (1S4), another showed FR with Pdr1p N1086I (1S3), and two were F-SDD with no Pdr1p SNP (1S1 and 1S2). Patient 2 was admitted to the same NICU on day 17 of Patient 1’s hospitalization. Both patients were hospitalized concurrently for 87 days and were attended to by the same medical staff. Patient 2’s blood cultures on day 9 yielded both an F-SDD (2B1) isolate with no Pdr1p SNP and an FR (2B2) isolate with Pdr1p N1086I, but only an F-SDD strain had been reported by the clinical microbiology laboratory of the hospital A where the NICU is located. Five isolates (three blood, one stool, and one catheter) obtained during days 12–29 were F-SDD and did not harbor Pdr1p SNPs. However, three blood isolates obtained on days 33, 43, and 59 and one catheter isolate (2C2) on day 33 were FR, and they harbored Pdr1p P258L. Follow-up blood cultures taken after HD 62 were negative. All six serial isolates from Patient 3 were F-SDD and did not harbor SNPs for antifungal resistance genes despite a long course of treatment with fluconazole, amphotericin B, and caspofungin. All four stool cultures obtained during the period of positive blood cultures were negative for *C. glabrata*.

### Comparison of 48 blood isolates from different patients

Table 2 lists the antifungal susceptibility data, as well as the genetic characteristics obtained through WGS for the 48 BSI isolates of *C. glabrata* from 15 hospitals. Of 48 patient isolates, 27 (Isolates 4 to 30) were FR, while 21 (Isolates 31 to 51) were F-SDD. Upon exclusion of MLST genotype-specific Pdr1p SNPs, which presented in both F-SDD and FR isolates of the same ST, 23 of the 27 FR isolates of *C. glabrata* displayed FR-specific SNPs in Pdr1p, and four FR isolates (Isolates 15, 23, 26, and 30) had no Pdr1p SNP. The analysis of SNPs in representative *ERG* genes (*ERG 1–11*, *ERG 13*, *ERG 20*, and *ERG 25–27*) revealed that seven isolates (Isolates 11, 13, 19, 26, 28, 32, and 33) had SNPs in one of the five *ERG* genes (*ERG* 2, *ERG* 3, *ERG* 4, *ERG* 11, or *ERG* 20) upon exclusion of MLST genotype-specific SNPs. Only one isolate (Isolate 13) had an *ERG11* SNP (Erg11p K108N), while remaining 47 isolates did not exhibit *ERG11* SNPs. All the isolates were susceptible to amphotericin B (≤ 2 µg/mL) as determined by the Sensititre YeastOne system. However, upon reanalysis of amphotericin B MICs using Etest (BioMerieux, France) for these seven isolates, one isolate (Isolate 11) with the Erg2 A84fs mutation showed a higher amphotericin B MIC of 3 mg/mL (Table 2). Among the 48 BSI isolates of *C. glabrata*, six (Isolates 11–14, 17, and 37) exhibited resistance to one of three echinocandins and had SNPs in the hotspot region of *FKS1* or *FKS*2. Two isolates (11 and 13) that were resistant to the three echinocandins had other SNPs outside the *FKS2* hotspots, and one isolate (Isolate 27) with a caspofungin MIC of 0.25 µg/mL (intermediate) had SNPs outside the *FKS1* hotspots. The other seven isolates (4, 8, 30, 41, 48, 50, and 51) with SNPs outside the *FKS1* or *FKS2* hotspots were susceptible to the three echinocandins. The 48 isolates tested had low MICs against 5-FC (≤ 0.06 µg/mL) and no SNP in genes associated with 5-FC resistance.

**TABLE 2:** Antifungal susceptibilities and whole-genome sequence-based molecular characteristics of 48 bloodstream isolates of *C. glabrata* from 48 patients in 15 hospitals, collected as part of a South Korean multicenter surveillance program^*d*^

Isolate (patient) no.	Antifungal minimum inhibitory concentrations (ug/mL)^*a*^					Results of whole-genome sequencing^*b*^					
	FLU	AMB (Etest)	ANF	CAF	MCF	MLST (ST)	Pdr1p	All Erg	Fks1p	Fks2p	Msh2p
4	**128**	1	0.12	0.12	0.015	3	A731E			E291V	
5	**256**	1	0.12	0.12	0.015	3	Y584D				
6	**256**	1	0.12	0.12	0.015	3	T1080N				
7	**64**	1	0.06	0.12	0.015	3	F832_L833delinsLV				
8	**128**	1	0.12	0.12	0.03	3	Y1106N			T1165I	
9	**256**	1	0.03	0.12	0.015	3	S942F				
10	**64**	1	0.12	0.12	0.015	7	Y1106*/S236N				V239L
11	**64**	**2 (3**)	**2**	**8**	**4**	7	L347F	Erg2p A84fs	**S629P**	W1530R	V239L
12	**128**	1	**0.5**	**1**	0.06	7	E340G			**F659S**	V239L
13	**64**	1 (0.06)	**1**	**2**	**0.25**	7	Y556C	Erg11p K108N	**P633T**	S1293P**/D666N**	V239L
14	**256**	0.5	**0.5**	**1**	**0.25**	7	R376Q			**S663P**	V239L
15	**64**	1	0.03	0.06	0.015	7	None				V239L
16	**256**	1	0.06	0.12	0.015	7	N1086Y				V239L
17	**128**	1	0.06	**0.5**	0.06	7	A731E			**F659S**	V239L
18	**128**	1	0.06	0.12	0.015	22	Y932C				E456D
19	**256**	1 (0.5)	0.12	0.12	0.015	26	S316I	Erg4p T140A			
20	**128**	1	0.12	0.12	0.015	26	F377I				
21	**128**	1	0.12	0.12	0.015	26	N1091D				
22	**256**	1	0.06	0.12	0.015	26	E388Q				
23	**64**	1	0.03	0.06	0.015	26	None				
24	**256**	1	0.12	0.12	0.015	26	K365E				
25	**256**	1	0.12	0.12	0.03	26	R376Q				
26	**64**	0.5 (0.5)	0.03	0.06	0.015	26	None	Erg4p T140A			
27	**256**	1	0.12	0.25	0.03	55	P258S		F68L		
28	**64**	1 (0.5)	0.25	0.25	0.03	55	F294S	Erg3p W98*			
29	**128**	1	0.12	0.12	0.015	59	E369K				
30	**64**	0.25	0.06	0.06	0.015	59	None		G75D		
31	16	1	0.06	0.12	0.015	3	None				
32	16	1 (0.5)	0.06	0.12	0.015	3	None	Erg20p D146E			
33	16	1 (0.25)	0.06	0.12	0.015	3	None	Three SNPs^*c*^			
34	8	1	0.06	0.12	0.015	3	None				
35	16	2	0.03	0.06	0.015	3	None				
36	8	1	0.12	0.12	0.015	7	None				V239L
37	8	0.5	0.25	**0.5**	0.06	7	None			**F659Y**	V239L
38	32	0.5	0.03	0.06	0.015	7	None				V239L
39	16	0.5	0.12	0.12	0.03	7	None				V239L
40	16	2	0.015	0.03	0.008	7	None				V239L
41	32	0.5	0.03	0.06	0.015	22	None		Y3D		E456D
42	16	1	0.06	0.12	0.015	26	None				
43	16	1	0.06	0.12	0.015	26	None				
44	16	1	0.06	0.12	0.015	26	None				
45	16	1	0.06	0.12	0.015	26	None				
46	16	1	0.06	0.12	0.015	26	None				
47	16	1	0.06	0.06	0.015	26	None				
48	16	1	0.06	0.12	0.015	26	None		A1453V		
49	16	1	0.06	0.12	0.015	26	None				
50	32	0.5	0.03	0.06	0.015	55	None			Q12K	
51	16	1	0.06	0.12	0.015	55	None			K1867N/Q12K	

^*a*^ Antifungal minimum inhibitory concentrations were determined using the Sensititre YeastOne system (Thermo-Scientific, Cleveland, OH, USA). The antifungal resistance category (fluconazole and three echinocandins) and non-wild-type (amphotericin B) were identified using the Clinical and Laboratory Standards Institute (CLSI) M60-ED (8) and the proposed SYO-specific epidemiological cutoff values (19), respectively. Bold indicates antifungal-resistant or non-wild-type isolates.

^*b*^ The listed SNPs in Pdr1p, all Erg (*ERG1–ERG11*, *ERG13*, *ERG20*, and *ERG25–ERG27*), and Fks1p or Fks2p were obtained after eliminating MLST genotype-specific SNPs (Table S2). There were no SNPs in the genes associated with 5-FC resistance (*FCY1*, *FCY2*, *FPS1*, and *FPS2*) in any of the isolates.

^*c*^ Isolate 33 had three SNPs, Erg5p G286D, Erg7p V280M, and Erg10p K191Q.

^*d*^ FLU, fluconazole; AMB, amphotericin B, ANF, anidulafungin; CAF, caspofungin; MCF, micafungin; MLST, multilocus sequence typing; ST, strain type.

### Nosocomial cluster of FR *C. glabrata* BSI in five patients

**Fig 4:**
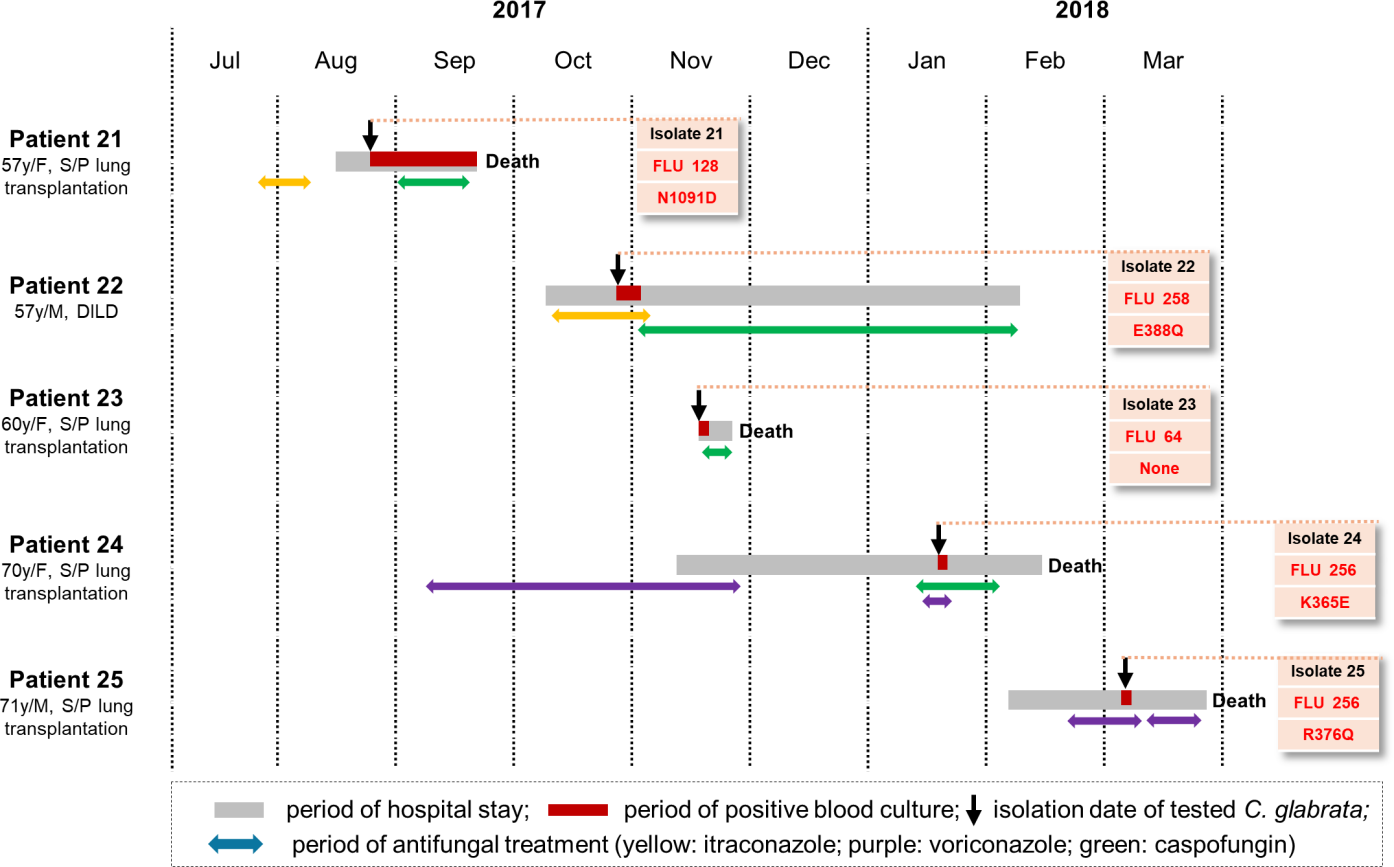
Schematic of the epidemiological status, antifungal treatment regimens, and outcomes of five patients (Patients 21 to 25) with clonal fluconazole-resistant bloodstream isolates of *C. glabrata* obtained between August 2017 and March 2018. Boxes contain isolate numbers with fluconazole minimum inhibitory concentrations (MICs, mg/L) and corresponding Pdr1p single-nucleotide polymorphisms (SNPs). See Table 2 for information on the isolates. Abbreviations: y, year; F, female; M, male; S/P, status post; DILD, diffuse interstitial lung disease; FLU, fluconazole.

Figure 4 shows the epidemiological status, antifungal treatments, and outcomes of five patients with *C. glabrata* fungemia (Patients 21–25), whose FR blood isolates formed a clonal cluster in the WGS SNP-based phylogenetic tree. Between August 2017 and March 2018, five isolates were obtained from five patients who were admitted to the pulmonary department of hospital B. Of the five patients, four (Patients 21 and 23–25) had undergone lung transplantation, and one (Patient 22) had diffuse interstitial lung disease. All the patients were hospitalized in the ICU and had central venous catheters when the initial positive culture results were obtained. Notably, the isolates from four patients (Patients 21, 22, 24, and 25) who had previously received azole antifungal therapy showed Pdr1p SNPs (N1091D, E388Q, K365E, and R376Q, respectively), and the isolate (Isolate 23) from Patient 23, who had not previously received azole antifungal therapy, had no Pdr1p SNP. All the patients received antifungals such as caspofungin (Patients 21–24) or voriconazole (Patient 25), and the four patients (Patients 21, 22, 24, and 25) who had undergone lung transplantation died.

### SNPs of candidate genes associated with azole resistance

**Fig 5:**
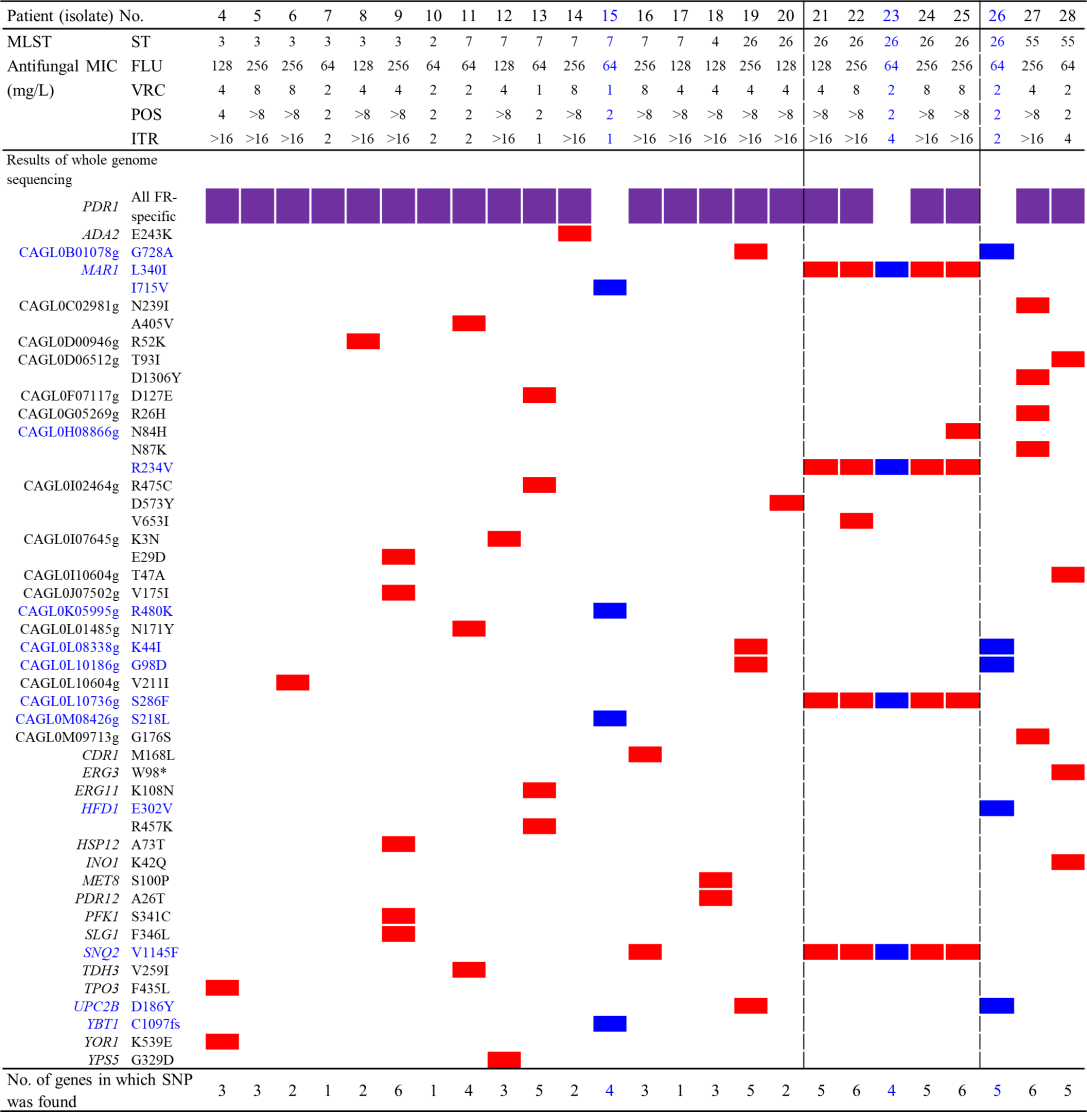
Single-nucleotide polymorphisms (SNPs) in genes potentially linked to azole resistance in fluconazole-resistant bloodstream isolates of *C. glabrata* by whole-genome sequencing (WGS). Three isolates resistant to fluconazole (Isolates 15, 23, and 26) that did not have the Pdr1p SNP and their associated nucleotide polymorphisms in other potential azole-resistant genes are in blue text or blue boxes. Five isolates (Isolates 21 to 25) that formed a clonal cluster in a phylogenetic tree based on WGS SNP analysis are in the large black box. Abbreviations: MLST, multilocus sequence typing; ST, strain type; FLU, fluconazole; VRC, voriconazole; POS, posaconazole, ITR, itraconazole.

Figure 5 shows the SNPs in 39 candidate genes associated with azole resistance in 25 FR (Isolates 4–28) isolates of *C. glabrata*. Initially, 182 genes associated with potential antifungal resistance were examined in 27 FR isolates (Isolates 4–30), and their SNPs were compared to those of 21 F-SDD isolates (Isolates 31 to 51). SNPs present among both FR and F-SDD isolates of the same ST were excluded as they were considered MLST-specific. Two FR ST 59 isolates (Isolates 29 and 30) were not included due to the lack of control F-SDD isolates from ST 59. Among 182 genes, SNPs were found in 48 genes of 25 FR isolates (Isolates 4–28). After consulting the *Candida* Genome Database (http://candidageome.org/), nine genes possibly unrelated to azole resistance (*FKS1*, *FKS2*, *FKS3*, *IFA38*, *SSD1*, *ERG2*, *FLR2*, *FPS1*, and *FPS2*) were eliminated, leaving 39 candidate genes associated with azole resistance. Overall, by excluding MLST-specific SNPs and subsequently SNPs in F-SDD isolates, we identified FR-specific SNPs on 12 genes in three *PDR1*-negative FR isolates: four SNPs in Isolate 15, four SNPs in Isolate 23, and five SNPs in isolate 26. Two ST 59 isolates (Isolates 29 and 30) were excluded because both were FR. Notably, the same SNPs in four azole-associated genes (Mar1p L340I, CAGL0H08866g R234V, CAGL0L10736g S286F, and SNQ2 V1145F) were shared by five FR BSI isolates of *C. glabrata* (Isolates 21–25), which formed a cluster in the phylogenetic analysis.

## DISCUSSION

Various methods for genotyping of *C. glabrata* have been used, such as microsatellite typing, pulsed-field gel electrophoresis, MLST, and WGS (21–24, 30–34). Among these, MLST has been widely utilized as the genotyping technique for *C. glabrata* (32–34). In two previous WGS studies that examined *C. glabrata* isolates from various geographic locations and body sites, *C. glabrata* isolates were clustered phylogenetically based on their various MLST genotypes regardless of their resistance profile or origin (25, 26). Our phylogenetic analysis based on the ANI from WGS data found that the 79 isolates of *C. glabrata* could be classified according to their six MLST genotypes. Furthermore, we identified identical SNPs in 128 genes potentially associated with antifungal resistance, regardless of their antifungal susceptibility within the same ST (MLST-specific SNPs). C. *glabrata* BSI isolates from a particular geographic area have been reported to include a relatively small number of major STs by MLST analysis, but *C. glabrata* strains sharing identical STs have been identified across various geographic areas (25, 35, 36). Therefore, we conducted a detailed examination of the WGS phylogenetic tree within the identical MLST isolates in combination with epidemiological data to detect nosocomial transmission of *C. glabrata* within a single hospital over a period of several months.

The development of *C. glabrata* BSI has been considered to be exclusively attributable to autoinfection by endogenous *C. glabrata* strains initially colonizing the mucosa of the gastrointestinal tract, but some recent studies suggest an epidemiological link between certain BSI isolates of *C. glabrata,* indicating that infection can occasionally spread among patients (21, 22). In a recent WGS study using 80 clinical isolates of *C. glabrata* collected from various body sites in six different hospitals in Qatar, several putative clonal outbreaks were detected, suggesting persistent clonal strains of *C. glabrata* within and among hospitals (24). In the present study, we analyzed 31 (15 FR and 16 F-SDD) epidemiologically related *C. glabrata* isolates from three premature infants in a single NICU in 2022, in comparison with 48 (27 FR and 21 F-SDD) unrelated *C. glabrata* BSI isolates from 15 hospitals during 2010–2022 as control strains to trace the possible transmission patterns of clonal FR *C. glabrata* isolates via WGS. Phylogenetic analysis showed two epidemiologically related clusters of ST26 isolates: one (Cluster 1) consisting of 15 FR and 10 F-SDD isolates obtained from two infants with overlapping NICU hospitalizations over 3 months, among which the FR isolates harbored two different Pdr1p SNPs, and another (Cluster 2) consisting of five FR isolates from five patients at the same hospital over 8 months. All five patients had received care in the same pulmonary department, and FR isolates from four patients who received azole therapy had four different Pdr1p SNPs. These results indicate the transmission of clonal *C. glabrata* BSI strains with different levels of fluconazole susceptibility or distinct Pdr1p SNP profiles among patients receiving antifungal therapy.

WGS has shown that the variation in SNPs within a patient can be used as a reference point to determine whether the transmission of *C. glabrata* occurred between a donor and a host or was acquired within a healthcare setting (37). However, comparing SNP cutoffs between studies is hampered by differences in bioinformatics methods and reference genomes, which can affect the number of SNPs identified (24, 37). In addition, serial isolates of *C. glabrata* from the same patient revealed marked genomic differences, including point mutations, insertions, deletions, and whole or partial chromosomal aneuploidies, which may have been affected by antifungal therapy (10, 38). In this study, we examined the total number of SNPs in a 12.3-Mb region. The SNP differences between isolates from the same patients (Patient 1 or 2) were similar to those between isolates from two clonal clusters (Clusters 1 and 2). It is worth noting that we observed significantly lower SNP variance between isolates from two clonal clusters (Clusters 1 and 2), with both cluster isolates being ST 26, in comparison with consecutive ST3 isolates from Patient 3.

Although transmission of *C. glabrata* BSI in newborns in a hospital setting is rare (39), our findings confirm that *C. glabrata* BSI was transmitted between two premature infants in the same NICU. Stool cultures from Patient 1 on hospital day 38 showed both F-SDD isolates without Pdr1p SNPs and FR isolates with Pdr1p N1086I or Pdr1p P258L. These strains were also found in blood, stool, and catheter cultures of Patient 2. These findings imply that the gastrointestinal tract functions as a reservoir for the dissemination of these strains, as we reported previously (10), leading to persistent fungemia in both patients for 51 and 56 days, respectively, despite prolonged antifungal therapy. A recent WGS study demonstrated that the blood cultures of contemporaneous strains of *C. glabrata* contained mixed populations of clonal but genetically diverse strains (38). Genetically distinct strains from the same patient exhibited mixed F-SDD and FR populations, but the clinical microbiology laboratory only identified an F-SDD index strain. Similarly, the first blood cultures in our Patient 2 were a mixture of an F-SDD isolate (2B1) with no Pdr1p SNP and an FR (2B2) isolate with Pdr1p N1086I, but the clinical microbiology laboratory only identified an F-SDD strain. Five isolates (three blood, one stool, and one catheter) from Patient 2 obtained during days 12–29 were F-SDD, while three blood and one catheter isolates obtained on days 33–59 were FR, and they harbored Pdr1p P258L. Given that the fitness cost related to acquisition of azole resistance by *C. glabrata* is unclear, further studies are needed to determine how the intra-patient mutation rate of *C. glabrata* affects nosocomial transmission.

Unexpectedly, we found another clonal cluster of five FR BSI isolates of *C. glabrata* from five patients (Patients 21–25) at the same hospital over 8 months. Of the five patients, four had undergone lung transplantation and one had diffuse interstitial lung disease. Among these five FR isolates, one had no Pdr1p mutation but four obtained after exposure to azole antifungals harbored distinct Pdr1p SNPs (N1091D, E388Q, K365E, and R376Q). One possibility is that two or more clonal strains of *C. glabrata* may acquire resistance to azole antifungals through different mutation sites in the same Pdr1 gene despite sharing the same genetic background. Alternatively, a previous *in vitro* study demonstrated that single wild-type or laboratory strains of *C. glabrata* could produce four or six distinct Pdr1p mutants, respectively, after exposure to fluconazole-containing medium (40), which supports the clonality of our five FR isolates with distinct Pdr1p SNP patterns.

In hospital B in which we found Cluster 2, *C. glabrata*, predominantly FR, made up 36.5% of all *Candida*-positive respiratory samples in the setting of azole administration for 6 months in patients post-lung transplantation, followed by *C. albicans,* at 21.2% (41). The overall cumulative incidence of *Candida* species was approximately threefold that of *Aspergillus* species (41). It has been proposed that underlying lung conditions, prior use of antifungal agents before transplantation, an extended hospital stay after transplantation, and advanced age are linked to the development of fungal infections, significantly heightening the risk of treatment failure and overall mortality after lung transplantation (41); this is reflected in our findings. We speculate that the sporadic occurrence of *C. glabrata* BSI cases, the intervals between the five cases, and the transmission leading to respiratory colonization without subsequent BSI may hamper the identification of clonal transmission of FR *C. glabrata* BSIs. Overall, these findings emphasize the importance of the awareness of the possibility of clonal dissemination of fluconazole-resistant *C. glabrata* isolates in clinical settings where azole usage is frequent, as suggested by others (21).

WGS has been used to identify the potential genetic markers for antifungal resistance or genetic factors underlying antifungal resistance in *C. glabrata* (10, 25–27). In this WGS analysis, the six isolates resistant to at least one echinocandin had SNPs in the hotspot region of *FKS1* or *FKS2*, both of which are associated with echinocandin resistance (6). Acquired amphotericin B resistance in *Candida* isolates is uncommon, and only the Etest is predictive of amphotericin B resistance (10, 42). *ERG2, ERG3, ERG4*, and *ERG6* have been implicated in the mechanism of amphotericin B resistance (3, 10, 43). In this study, one isolate with an amphotericin B MIC of 3 mg/L by Etest had the Erg2p A84fs mutation, but further work is needed to confirm its importance.

This study had some limitations, including the fact that the isolates used for WGS did not represent the main MLST genotypes in Korea, which are predominantly ST3 and ST7 (70% of isolates) (35). Our initial experiments revealed that isolates from two NICU infants belonged to ST26, prompting us to include more ST26 isolates for comparison. In addition, several FR-specific Pdr1p SNPs detected in this study did not confirm their roles in resistance, although our previous study showed that one or two additional Pdr1p SNPs, except MLST-specific Pdr1p SNPs, were found in 98.5% (65/66) of FR isolates and only 0.9% (2/212) of F-SDD isolates (8). Furthermore, we identified 13 candidate FR-specific SNPs of 12 genes in three FR isolates without Pdr1p SNPs, all of which were novel. Determining the function of each SNP in genes associated with azole resistance among unmatched isolates is challenging, and further research is necessary to characterize these SNPs and their functions in the development of azole resistance.

## MATERIALS AND METHODS

### Fungal isolates and identification

A total of 79 clinical isolates of C. glabrata from 51 patients in 15 South Korean hospitals between 2010 and 2022 were analyzed. The collection was composed of a subset of 31 strains (20 blood, six stool, and five catheter isolates) isolated from three premature infants (Patients 1 to 3) in a single NICU during the same period in 2022, along with 48 unrelated strains obtained from blood cultures of 48 patients (Patients 4 to 51) spanning a 12-year period (2010–2022) across 15 hospitals (A to O) nationwide. The NICU, located at Chonnam National University Hospital (CNUH) in Gwangju, South Korea, is an open room with 45 patient beds. CNUH (hospital A) is a 1,050-bed teaching hospital and a tertiary care referral center. Α total of 48 control isolates were selected from BSI isolates of *C. glabrata* with documented antifungal susceptibility and MLST genotypes in the Korean collection, which were obtained through a multicenter surveillance program conducted in South Korea (7, 8). Species identification was performed using matrix-assisted laser desorption/ionization time-of-flight mass spectrometry (Biotyper; Bruker Daltonics, https://www.bruker.com/) with library version 4.0 or by sequencing of the D1/D2 domains of the 26S rRNA gene (8). Retrospective collection of clinical information included patient demographics, diagnosis, clinical status at the time of positive culture, and therapeutic modalities (8). The study received approval from the Ethics Committee of CNUH. The requirement for informed parental consent was waived (CNUH-2020–117).

### Antifungal susceptibility testing

The minimum inhibitory concentrations (MICs) for fluconazole, voriconazole, posaconazole, itraconazole, amphotericin B, anidulafungin, caspofungin, micafungin, and 5-FC were determined using the Sensititre YeastOne system (Thermo Fisher Scientific, Waltham, MA, USA). For some isolates, the antifungal MICs of amphotericin B (AMB) were also analyzed using Etest (bioMérieux, Marcy-l’Étoile, France). Each antifungal susceptibility test included two reference strains, *C. parapsilosis* ATCC 22019 and *Candida krusei* ATCC 6258, as quality control isolates. The MIC interpretive criteria consisted of species-specific Clinical and Laboratory Standards Institute (CLSI) clinical breakpoints for fluconazole (resistant, ≥64 mg/L; susceptible dose-dependent, ≤32 mg/L), anidulafungin (resistant, ≥0.5 mg/L; intermediate, 0.25 mg/L), caspofungin (resistant, ≥0.5 mg/L; intermediate, 0.25 mg/L), and micafungin (resistant, ≥0.25 mg/L; intermediate, 0.12 mg/L) (28). The MIC interpretive criteria for amphotericin B (susceptible, ≤2 mg/L; resistant, >2 mg/L), voriconazole (resistant, >2 mg/L), posaconazole (resistant, >4 mg/L), and itraconazole (resistant, >2 mg/L) were based on the proposed SYO-specific epidemiological cutoff values (ECVs) (29).

### WGS analysis

DNA was extracted from 79 isolates of *C. glabrata* using QIAamp DNA Mini Kits (Qiagen, Aarhus, Denmark) according to the manufacturer’s protocol. Subsequently, a library was prepared using an Illumina DNA Flex Kit (Illumina, San Diego, CA, USA), and sequencing was performed as 150-bp paired ends using a NovaSeq 6000 system (Illumina). All steps in the subsequent WGS data analysis and inspection of the accuracy of variant calling were conducted using the tools in CLC Genomics Workbench version 20.0.4 (Qiagen, Aarhus, Denmark) with the default parameters, unless otherwise stated. The sequences were trimmed using the “Trim reads” tool (quality trim, 0.05; ambiguous limit, 2). The trimmed reads were mapped to the *C. glabrata* reference genome (CBS 138) from the Candida Genome Database (http://www.candidagenome.org/) using the “Map Reads to Reference” tool (length fraction, 0.5; similarity fraction, 0.8). The “Local realignment” tool was applied to improve the alignments around insertions and deletions (indels). For variant calling of genes associated with antifungal resistance, the “Basic Variant Detection” tool was used with a parameter of minimum coverage, 10; minimum count, 2; and minimum frequency, 35.0%. The accuracy of variant calling was visually inspected using sequence viewer in CLC Genomics Workbench.

### Phylogenetic analysis based on WGS and MLST

All processes of the phylogenetic analysis based on WGS and MLST were conducted using the tools in the CLC Genomics Workbench (44, 45). Following “WGS analysis” described above, whole-genome sequences of 79 *C*. *glabrata* isolates obtained from WGS and whole-genome sequence of Australian *C. glabrata* strain WM_18.66 (used as the outgroup of phylogenetic analysis, downloaded from NCBI Sequence Read Archive: BioProject PRJNA480138) were trimmed, mapped to the reference genome (CBS138), and realigned to the generated realigned sequences (25, 44). Consensus sequences (12,316,909–12,317,854 bp) of 79 *C*. *glabrata* isolates and WM_18.66 were constructed from realigned sequences using “Extract Consensus Sequence.” Consensus sequences of 79 *C*. *glabrata* isolates were aligned with CBS138 and WM_18.66 with the “Whole Genome Alignment” tool (minimum initial seed length 15; minimum alignment block length 100), and the aligned output was entered into the “Average Nucleotide Comparison” tool (minimum similarity fraction 0.8, minimum length fraction 0.8) to quantify the similarity between genomes. The aligned regions for each pair of genomes were identified to calculate the ANI, defined as the average percentage of matching nucleotides within these aligned regions. The pairwise comparison table generated with the comparison tool was entered into the “Tree from Comparison” tool for construction of an NJ tree (44, 45). For MLST, performed *in silico*, sequences were extracted from six genes (*FKS2*, *LEU2*, *NMT1*, *TRP1*, *UGP1*, and *URA3*) within the consensus sequences generated by the method described previously (46, 47). The sequences of six genes were searched in the pubMLST webtool (http://www.pubmlst.org/) to determine the ST. Subsequently, the sequences of the six genes were concatenated into a single sequence of 3,345 bp. These concatenated sequences were then aligned using the “Create alignment” tool, and a phylogenetic tree was constructed using the NJ method based on Kimura 80 in the “Create tree” tool. Isolates forming clusters in the phylogenetic tree were carefully inspected, and their epidemiological data (e.g., isolation years and hospitals) and clinical information were reviewed. If epidemiologically related strains from two or more patients had high genetic similarity and were distinct from other isolates in phylogenetic analysis of WGS data, they were defined as isolates of a possible nosocomial cluster (24).

### Analysis of SNP differences

SNP differences between isolates at 12,338,304 nucleotide positions of the genome were calculated using the MEGA-X pairwise distance matrix (37, 48). For input in the calculation of SNP differences, alignment using BWA (version 0.7.17), variant calling using GATK (version 4.4.0.0) HaplotypeCaller with the option “QD  < 2.0, FS  > 60.0, MQ  < 40.0, SOR  >  3.0, MQRankSum  < −12.5, and ReadPosRankSum  < −8.0” for filtration, and concatenation using GATK FastaAlternateReferenceMaker were performed. Differences in SNP density between isolates were calculated by dividing the SNP difference by the total number of nucleotide positions (kb) in the genome.

### Analysis of antifungal resistance-specific mutations

Nonsynonymous mutations including SNPs and indels linked to resistance to antifungal drugs and their correlations with antifungal MICs were investigated. We first analyzed the nonsynonymous mutations of all genes among isolates that belonged to the same MLST genotype to eliminate MLST-specific SNPs. Subsequently, we compared these mutations with those in antifungal-susceptible or F-SDD isolates to identify candidate antifungal resistance-specific or FR-specific mutations. For all isolates, mutations in genes associated with resistance to azole drugs (*PDR1*), echinocandins (*FKS1* and *FKS2*), 5-fluorocytosine (*FCY1*, *FCY2*, *FPS1*, and *FPS2*), and representative *ERG* (*ERG 1–11*, *ERG 13*, *ERG 20*, and *ERG 25–27*) and *MSH2* genes were examined (10, 49–51). For FR isolates, nonsynonymous mutations in 182 genes identified previously (10, 52) were examined among the same MLST genotype isolates, and mutations in genes with FR-specific SNPs were analyzed for their associations with azole resistance using information from the Candida Genome Database (http://www.candidagenome.org/). After excluding MLST genotype-specific Pdr1p SNPs, which were present in both F-SDD and FR isolates of the same ST (as shown in Table S2), the remaining Pdr1p SNPs that were exclusively present in FR isolates were categorized as FR-specific SNPs.

### Statistical analysis

To compare median SNP differences among ST groups and subgroups, the Kruskal–Wallis test followed by nonparametric multiple comparisons using the R-nparcomp package in RStudio version 2022.7.1.554 (RStudio Inc., Boston, MA, USA) and box plots were generated using Prism version 10.2.1 (GraphPad Software, San Diego, CA, USA) (48). In all analyses, *P* < 0.05 was taken to indicate statistical significance.

## Data Availability

The raw sequence data have been deposited in the NCBI Sequence Read Archive (BioProject PRJNA1074512).

## SUPPLEMENTAL MATERIAL

Figure S1 spectrum.00883-24-s0001.tif SNP differences between isolates.

Table S1 and S2 spectrum.00883-24-s0002.docx Table S1: The 128 candidate genes associated with antifungal resistance in *C. glabrata*. Table S2: SNPs in representative genes.

ASM does not own the copyrights to Supplemental Material that may be linked to, or accessed through, an article. The authors have granted ASM a non-exclusive, world-wide license to publish the Supplemental Material files. Please contact the corresponding author directly for reuse.
