# Supplementary figures and images for: Nosocomial transmission of fluconazole-resistant Candida glabrata bloodstream isolates revealed by whole-genome sequencing
Source: Microbiol Spectr. 2024 Aug 20;12(10):e00883-24. doi: 10.1128/spectrum.00883-24 (PMC11448407; doi:10.1128/spectrum.00883-24)

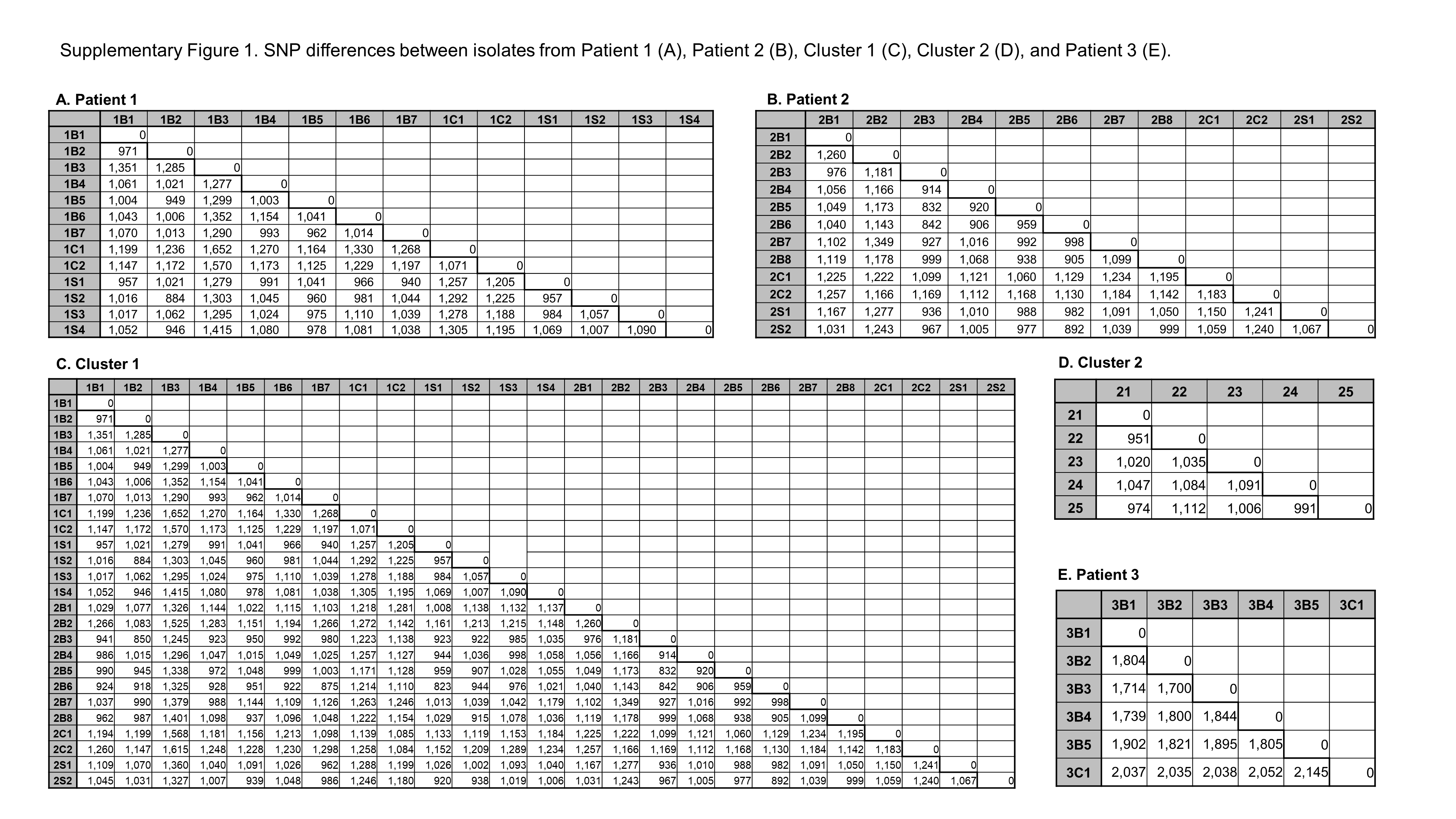

Supplement: Figure S1 — SNP differences between isolates. [file spectrum.00883-24-s0001.tif]
