# Supplementary material for: Nosocomial transmission of fluconazole-resistant Candida glabrata bloodstream isolates revealed by whole-genome sequencing
Source: Microbiol Spectr. 2024 Aug 20;12(10):e00883-24. doi: 10.1128/spectrum.00883-24 (PMC11448407; doi:10.1128/spectrum.00883-24)
Supplement: Table S1 and S2 — Table S1: The 128 candidate genes associated with antifungal resistance in C. glabrata. Table S2: SNPs in representative genes. [file spectrum.00883-24-s0002.docx]

| **Supplemental Table S1.** The 128 candidate genes associated with antifungal resistance in *C. glabrata* | | | | | | | | | | |
| --- | --- | --- | --- | --- | --- | --- | --- | --- | --- | --- |
| Possible antifungal resistance genes | | | | | | | | | | |
| *ADA2* | *CAGL0B03355g* | *CAGL0G07689g* | *CAGL0K03377g* | *CAGL0L10318g* | *EPA3* | *FCY2* | *INO1* | *PDR13* | *SSD1* | *YOR1* |
| *ADH1* | *CAGL0B03421g* (*Mar1*) | *CAGL0G09273g* | *CAGL0K04301g* | *CAGL0L10604g* | *ERG1* | *FEN1* | *IPC1* | *PFK1* | *STB5* | *YPS5* |
| *ADK1* | *CAGL0C02981g* | *CAGL0G09603g* | *CAGL0K05995g* | *CAGL0L10736g* | *ERG2* | *FKS1* | *JJJ1* | *PGK1* | *STR3* |  |
| *AHP1* | *CAGL0D00946g* | *CAGL0H02387g* | *CAGL0K08866g* | *CAGL0M01870g* | *ERG3* | *FKS2* | *MEC3* | *PUP1* | *SUR2* |  |
| *AMT1* | *CAGL0D06512g* | *CAGL0H05181g* | *CAGL0K09460g* | *CAGL0M04631g* | *ERG4* | *FKS3* | *MET8* | *PYC1* | *SUR4* |  |
| *AP1* | *CAGL0E00803g* | *CAGL0H06809g* | *CAGL0K09702g* | *CAGL0M05005g* | *ERG5* | *FLR1* | *MGE1* | *QDR2* | *SUT1* |  |
| *AQR1* | *CAGL0E01353g* | *CAGL0H08866g* | *CAGL0K10934g* | *CAGL0M07766g* | *ERG6* | *FLR2* | *MSH2* | *ROX1* | *TDH3* |  |
| *ATF2* | *CAGL0E04554g* | *CAGL0I01980g* | *CAGL0K11616g* | *CAGL0M08426g* | *ERG7* | *FPS1* | *MT-II* | *RPN4* | *TOG1* |  |
| *BMT2* | *CAGL0E04576g* | *CAGL0I02464g* | *CAGL0K12100g* | *CAGL0M09713g* | *ERG8* | *FPS2* | *NCE103* | *RSB1* | *TPO1_1* |  |
| *BRE5* | *CAGL0F04917g* | *CAGL0I04422g* | *CAGL0K12958g* | *CAGL0M10219g* | *ERG9* | *GAS1* | *NDT80* | *RTA1* | *TPO1_2* |  |
| *CAGL0A01650g* | *CAGL0F05467g* | *CAGL0I07249g* | *CAGL0L01485g* | *CAGL0M12749g* | *ERG10* | *GLN3* | *NOP8* | *RTT106* | *TPO3* |  |
| *CAGL0A02816g* | *CAGL0F06897g* | *CAGL0I07645g* | *CAGL0L02211g* | *CAGL0M12925g* | *ERG11* | *GPD2* | *NUD1* | *SDH2* | *TPO4* |  |
| *CAGL0A04169g* | *CAGL0F07117g* | *CAGL0I10604g* | *CAGL0L03135g* | *CAGL0M14047g* | *ERG13* | *HAL9* | *OCH1* | *SEC53* | *UFD1* |  |
| *CAGL0A04543g* | *CAGL0G01122g* | *CAGL0J00363g* | *CAGL0L03223g* | *CAGL0M14091g* | *ERG20* | *HFD1* | *PCK1* | *SET1* | *UGP1* |  |
| *CAGL0A04829g* | *CAGL0G03861g* | *CAGL0J00451g* | *CAGL0L06864g* | *CDR1* | *ERG25* | *HSC82* | *PDC* | *SHM2* | *UPC2A* |  |
| *CAGL0B01078g* | *CAGL0G05269g* | *CAGL0J00891g* | *CAGL0L07678g* | *CTA1* | *ERG26* | *HSP12* | *PDH1* | *SLG1* | *UPC2B* |  |
| *CAGL0B01969g* | *CAGL0G06468g* | *CAGL0J07502g* | *CAGL0L08338g* | *ECM4* | *ERG27* | *HSP31* | *PDR1* | *SNQ2* | *VPH2* |  |
| *CAGL0B02343g* | *CAGL0G07271g* | *CAGL0K02563g* | *CAGL0L10186g* | *ENO1* | *FCY1* | *IFA38* | *PDR12* | *SPE3* | *YBT1* |  |
| The 128 shaded genes had MLST (ST3, ST7, ST22, ST26, ST55 and ST59)-specific SNPs. | | | | | | | | | | |
|  | | | |  |  |  |  |  |  |  |

**Supplementary Table S2**. Single-nucleotide polymorphisms (SNPs) in representative genes associated with antifungal resistance of *C. glabrata* found in all isolates of the same multilocus sequence type

| Genes^a^ | SNPs detected according to MLST strain type (number of isolates examined) | | | | | |
| --- | --- | --- | --- | --- | --- | --- |
|  | ST 3 (17) | ST 7 (13) | ST 22 (2) | ST 26 (41) | ST 55 (4) | ST 59 (2) |
| **Azole resistance** | |  |  |  |  |  |
| *PDR1* | D243N/L98S/V91I | T143P/L98S/V91I/S76P | T143P/L98S/V91I/S76P | T143P/L98S/V91I/S76P | T143P/L98S/V91I/S76P | T745A/T143P/L98S/V91I/S76P |
| **Echinocandin resistance** | |  |  |  |  |  |
| *FKS1* | G14S | None | None | None | None | None |
| *FKS2* | T926P | None | None | None | None | None |
| **Ergosterol biosynthesis pathway genes^b^** | |  |  |  |  |  |
| *ERG2* | I207V | I207V | I207V | I207V | I207V | I207V |
| *ERG4* | None | T13N | None | None | None | None |
| *ERG5* |  |  |  |  |  | V5I |
| *ERG6* | None | R48K | None | D172Y | R48K | None |
| *ERG7* |  | T732A |  | T732A | I497T/E668K/Q683L | D269N/I497T/T732A |
| *ERG8* | N448S | N448S | N448S | N448S/Q340K |  |  |
| *ERG9* | C344Y |  |  | C128F |  | K173T |
| *ERG10* | N170D | N170D | N170D | N170D | N170D | N170D |
| *ERG27* |  |  |  |  |  | Q220K/A23S |
| **5-Fluorocytosine resistance^b^** | |  |  |  |  |  |
| *FCY2* | None | None | None | A237T | A237T | None |
| *FPS1* | None | A316S | None | None | N27H | None |
| *FPS2* | N18D/H128N/A409T/K475R/D517Y | None | None | Q533H | N18D/A227S/A409T/K475R | N18D/A227S/A409T/K475R |
| **Multi-drug resistance** | |  |  |  |  |  |
| *MSH2* | None | V239L | E456D | None | None | None |

^a^ The *C. glabrata* reference genome (CBS 138) from the Candida Genome Database was used.

^b^ No MLST-specific SNP was found in *FCY1*, *ERG1*, *ERG3*, *ERG4*, *ERG11*, *ERG13*, *ERG20*, or *ERG26*.
